# Ethyl 3-[2-(3,4-dimeth­oxy­benz­yl)-1-phenyl­sulfonyl-1*H*-indol-3-yl]acrylate chloro­form hemisolvate

**DOI:** 10.1107/S1600536811017740

**Published:** 2011-05-20

**Authors:** M. Thenmozhi, T. Kavitha, V. Dhayalan, A. K. Mohanakrishnan, M. N. Ponnuswamy

**Affiliations:** a Centre of Advanced Study in Crystallography and Biophysics, University of Madras, Guindy Campus, Chennai 600 025, India; b Department of Organic Chemistry, University of Madras, Guindy Campus, Chennai 600 025, India

## Abstract

In the title compound, C_28_H_27_NO_6_S·0.5CHCl_3_, the ethyl acrylate substituent adopts an extented conformation with all torsion angles close to 180°. The chloro­form solvent mol­ecule is disordered across an inversion centre and is therefore half occupied. The mol­ecular packing is controlled by inter­molecular C—H⋯O inter­actions.

## Related literature

For general background to indoles, see: Hu *et al.* (2005 ▶); Nieto *et al.* (2005 ▶); Mathiesen *et al.* (2005 ▶); Olgen & Nebioglu (2002 ▶). For the sulfonyl moiety, see: Bassindale (1984 ▶). For hybridization, see: Beddoes *et al.* (1986 ▶). For hydrogen-bond motifs, see: Bernstein *et al.* (1995 ▶).
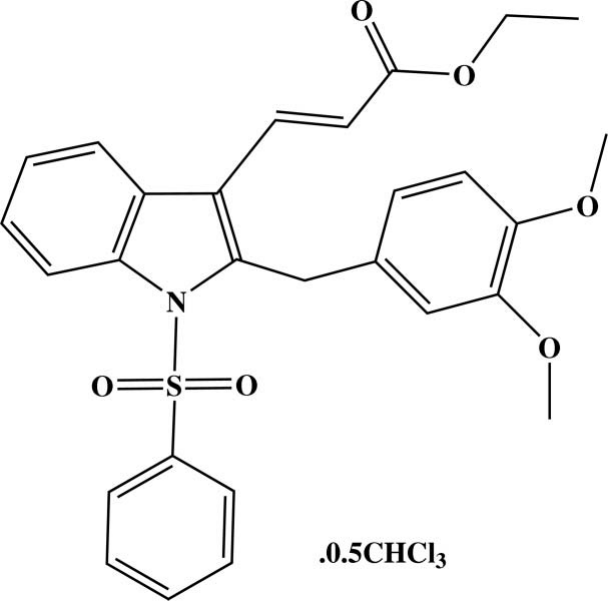


## Experimental

### 

#### Crystal data

C_28_H_27_NO_6_S·0.5CHCl_3_
*M* *_r_* = 565.25
Triclinic, 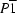

*a* = 10.2154 (3) Å
*b* = 12.0504 (4) Å
*c* = 12.6310 (4) Å
α = 70.602 (2)°
β = 69.613 (1)°
γ = 77.056 (2)°
*V* = 1364.39 (7) Å^3^
*Z* = 2
Mo *K*α radiation
μ = 0.31 mm^−1^
*T* = 293 K
0.30 × 0.25 × 0.20 mm

#### Data collection

Bruker Kappa APEXII area-detector diffractometer
Absorption correction: multi-scan (*SADABS*; Sheldrick, 2001 ▶) *T*_min_ = 0.913, *T*_max_ = 0.941
38797 measured reflections
10612 independent reflections
7709 reflections with *I* > 2σ(*I*)
*R*_int_ = 0.026

#### Refinement

*R*[*F* ^2^ > 2σ(*F* ^2^)] = 0.056
*wR*(*F* ^2^) = 0.189
*S* = 1.04
10612 reflections
364 parameters
27 restraints
H-atom parameters constrained
Δρ_max_ = 0.73 e Å^−3^
Δρ_min_ = −0.46 e Å^−3^

### 

Data collection: *APEX2* (Bruker, 2004 ▶); cell refinement: *SAINT* (Bruker, 2004 ▶); data reduction: *SAINT*; program(s) used to solve structure: *SHELXS97* (Sheldrick, 2008 ▶); program(s) used to refine structure: *SHELXS97* (Sheldrick, 2008 ▶); molecular graphics: *ORTEP-3* (Farrugia, 1997 ▶); software used to prepare material for publication: *SHELXL97* and *PLATON* (Spek, 2009 ▶).

## Supplementary Material

Crystal structure: contains datablocks global, I. DOI: 10.1107/S1600536811017740/bt5508sup1.cif

Structure factors: contains datablocks I. DOI: 10.1107/S1600536811017740/bt5508Isup2.hkl

Supplementary material file. DOI: 10.1107/S1600536811017740/bt5508Isup3.cml

Additional supplementary materials:  crystallographic information; 3D view; checkCIF report

## Figures and Tables

**Table 1:** Hydrogen-bond geometry (Å, °)

*D*—H⋯*A*	*D*—H	H⋯*A*	*D*⋯*A*	*D*—H⋯*A*
C15—H15⋯O4^i^	0.93	2.53	3.411 (3)	159
C24—H24*B*⋯O7^ii^	0.96	2.51	3.461 (2)	169
C24—H24*A*⋯O7^iii^	0.96	2.52	3.367 (3)	147
C30—H30*A*⋯O3^iv^	0.96	2.59	3.429 (7)	146
C30—H30*A*⋯O4^iv^	0.96	2.55	3.424 (7)	151

Symmetry codes: (i) 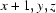; (ii) 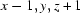; (iii) 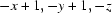; (iv) 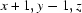.

**Fig. 1.**
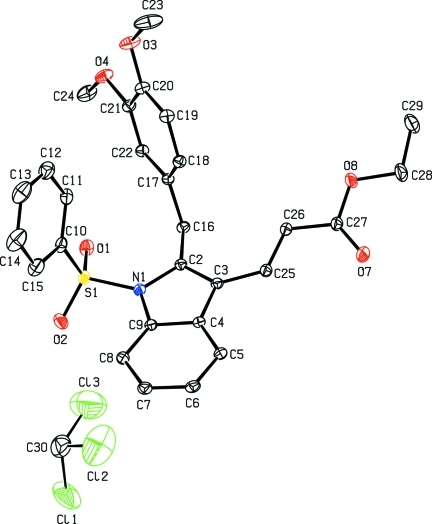
The ORTEP plot of the title compound, showing 30% probability displacement ellipsoids. H atoms omitted for clarity.

**Fig. 2.**
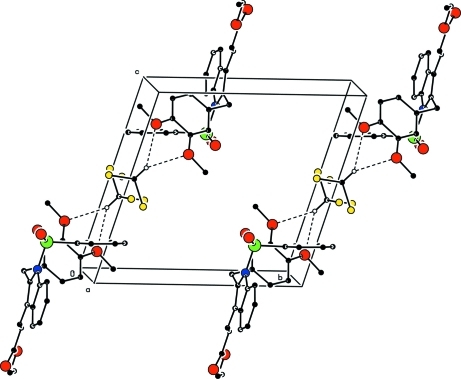
The packing of the molecules showing intermolecular C—H···O hydrogen bonds (dashed lines).

## supplementary crystallographic information

### Comment

Carboxylic acid indoles act as inhibitors of plasminogen activator inhibitor-1 (Hu *et al.*, 2005). The benzenesulfonamide group attached to the indole ring exhibit significant biological activities, such as antibacterial *etc*., (Nieto *et al.*, 2005). Also, the indole derivatives are identified as the molecules which interfere with a G protein-independent signaling pathway of the CRTH2 receptor (Mathiesen *et al.*,2005). N-substituted indole carboxylic acid esters have been prepared as possible *cyclo*-oxygenase-2 (COX-2) enzyme inhibitors (Olgen & Nebioglu, 2002).

Fig.1 shows the *ORTEP* plot of the title compound. The indole moiety is planar with the maximum deviation of -0.033 (2)Å and 0.028 (2)Å for atoms C5 and C7, respectively. The indole ring system is oriented equatorially to the phenyl sulfonyl ring [dihedral angle = 78.18 (6)°]. The O—S—O and N—S—C angles [See Table1] deviate significantly from their ideal value due to the Thrope-Ingold effect (Bassindale, 1984). The dimethoxybenzyl group is also equatorially oriented to the planar indole ring, which can be observed from the dihedral angle [78.04 (4)°].

The ethyl acrylate group substituted at C3 position of the indole ring takes up an extented conformation which is evident from the torsion angle values [C25—C26—C27—O8 =] -179.10 (16)°; [C25—C26—C27- O7 =] 1.8 (3)°; [C26—C27—O8—C28 =] 179.8 (2)°; [C29—C28—O8—C27 =] 179.7 (2)°. The sum of the bond angles around N1[359.37°] shows that atom N1 exhibits *sp*^2^ hybridization (Beddoes *et al.*, 1986). The chloroform solvent is disordered across inversion centre with the site occupancies of 0.5 for each.

The molecular packing is controlled by intra and intermolecular C—H···O interactions in addition to van der Waals forces (Table 2). Atom H15 of C15 form an intermolecular hydrogen bonding with atom O4, leading to a C11 linear chains (Bernstein *et al.*, 1995) with C—H···O type of interactions running along *a*-axis. The intermolecular interaction C24—H24A···O7 leads to *R*_2_^2^(13) dimers, which are connected through C24—H24B···O7 interaction as closed loops running along *c*-axis. Intermolecular bifurcated C—H···O hydrogen bonds involving the two oxygen atoms of the dimethoxybenzyl group and the hydrogen atom of the chloroform, solvent generates *R*_1_^2^(5) ring motif (Fig.2).

### Experimental

To a solution of (*E*)-Ethyl 3-(2-(bromomethyl)-1-(phenylsulfonyl)-1*H*-indol-3-yl) acrylate (0.3 g, 0.66 mmol) in dry DME (10 ml), ZnBr_2_ (0.3 g, 1.3 mmol), K_2_CO_3_ (0.18 g, 1.33 mmol) and veratrole (0.11 g, 0.79 mmol) were added. The reaction mixture was then refluxed for 12hrs under N_2_ atmosphere. The solvent was removed and the reaction mixture was quenched with ice-water (100 mL) containing 2 mL of conc.HCl. It was then extracted with chloroform (20 mL) and dried (Na_2_SO_4_). Removal of the solvent followed by crystallization from methanol afforded the product as pale yellow crystals.

### Refinement

H atoms were positioned geometrically (C—H = 0.93–0.97 Å) and allowed to ride on their parent atoms, with *U*_iso_(H) = 1.5*U*_eq_(C) for methyl and 1.2*U*_eq_(C) for other H atoms. The chloroform solvent is disordered across inversion centre with the site occupancies of 0.5 for each. The corresponding bond distances involving the disordered atoms C30, Cl1, Cl2 and Cl3 were restrained to 1.74 (1) Å and also their displacement parameters were restrained to an approximate isotropic behaviour.

### Crystal data

**Table:**

C_28_H_27_NO_6_S·0.5CHCl_3_	*Z* = 2
*M**_r_* = 565.25	*F*(000) = 590
Triclinic, *P*1	*D*_x_ = 1.376 Mg m^−^^3^
Hall symbol: -P 1	Mo *K*α radiation, λ = 0.71073 Å
*a* = 10.2154 (3) Å	Cell parameters from 10612 reflections
*b* = 12.0504 (4) Å	θ = 1.8–34.4°
*c* = 12.6310 (4) Å	µ = 0.31 mm^−^^1^
α = 70.602 (2)°	*T* = 293 K
β = 69.613 (1)°	Block, yellow
γ = 77.056 (2)°	0.30 × 0.25 × 0.20 mm
*V* = 1364.39 (7) Å^3^	

### Data collection

**Table:**

Bruker Kappa APEXII area-detector diffractometer	10612 independent reflections
Radiation source: fine-focus sealed tube	7709 reflections with *I* > 2σ(*I*)
graphite	*R*_int_ = 0.026
ω and φ scans	θ_max_ = 34.4°, θ_min_ = 1.8°
Absorption correction: multi-scan (*SADABS*; Sheldrick, 2001)	*h* = −15→16
*T*_min_ = 0.913, *T*_max_ = 0.941	*k* = −18→18
38797 measured reflections	*l* = −18→19

### Refinement

**Table:**

Refinement on *F*^2^	Primary atom site location: structure-invariant direct methods
Least-squares matrix: full	Secondary atom site location: difference Fourier map
*R*[*F*^2^ > 2σ(*F*^2^)] = 0.056	Hydrogen site location: inferred from neighbouring sites
*wR*(*F*^2^) = 0.189	H-atom parameters constrained
*S* = 1.04	*w* = 1/[σ^2^(*F*_o_^2^) + (0.1059*P*)^2^ + 0.3616*P*] where *P* = (*F*_o_^2^ + 2*F*_c_^2^)/3
10612 reflections	(Δ/σ)_max_ = 0.005
364 parameters	Δρ_max_ = 0.73 e Å^−^^3^
27 restraints	Δρ_min_ = −0.46 e Å^−^^3^

### Special details

**Table:**

Geometry. All e.s.d.'s (except the e.s.d. in the dihedral angle between two l.s. planes) are estimated using the full covariance matrix. The cell e.s.d.'s are taken into account individually in the estimation of e.s.d.'s in distances, angles and torsion angles; correlations between e.s.d.'s in cell parameters are only used when they are defined by crystal symmetry. An approximate (isotropic) treatment of cell e.s.d.'s is used for estimating e.s.d.'s involving l.s. planes.
Refinement. Refinement of *F*^2^ against ALL reflections. The weighted *R*-factor *wR* and goodness of fit *S* are based on *F*^2^, conventional *R*-factors *R* are based on *F*, with *F* set to zero for negative *F*^2^. The threshold expression of *F*^2^ > σ(*F*^2^) is used only for calculating *R*-factors(gt) *etc*. and is not relevant to the choice of reflections for refinement. *R*-factors based on *F*^2^ are statistically about twice as large as those based on *F*, and *R*- factors based on ALL data will be even larger.

### Fractional atomic coordinates and isotropic or equivalent isotropic displacement parameters (Å^2^)

**Table:**

	*x*	*y*	*z*	*U*_iso_*/*U*_eq_	Occ. (<1)
C2	0.72857 (13)	0.61852 (12)	0.05863 (12)	0.0264 (2)	
C3	0.81718 (13)	0.63351 (12)	−0.05351 (12)	0.0274 (2)	
C4	0.95289 (13)	0.65050 (12)	−0.05427 (12)	0.0263 (2)	
C5	1.07996 (14)	0.66763 (13)	−0.14331 (13)	0.0315 (3)	
H5	1.0889	0.6670	−0.2189	0.038*	
C6	1.19275 (15)	0.68563 (15)	−0.11662 (15)	0.0364 (3)	
H6	1.2784	0.6974	−0.1750	0.044*	
C7	1.17913 (15)	0.68629 (15)	−0.00368 (16)	0.0379 (3)	
H7	1.2559	0.7000	0.0116	0.045*	
C8	1.05530 (15)	0.66722 (15)	0.08678 (14)	0.0356 (3)	
H8	1.0476	0.6666	0.1625	0.043*	
C9	0.94241 (13)	0.64898 (12)	0.05935 (12)	0.0279 (2)	
C10	0.70544 (17)	0.78432 (16)	0.26272 (14)	0.0377 (3)	
C11	0.5742 (2)	0.84292 (17)	0.25639 (15)	0.0432 (4)	
H11	0.5084	0.8035	0.2523	0.052*	
C12	0.5421 (3)	0.9606 (2)	0.2561 (2)	0.0574 (5)	
H12	0.4541	1.0006	0.2523	0.069*	
C13	0.6404 (3)	1.0186 (2)	0.2616 (3)	0.0744 (8)	
H13	0.6187	1.0980	0.2609	0.089*	
C14	0.7695 (3)	0.9599 (3)	0.2680 (3)	0.0806 (9)	
H14	0.8348	0.9999	0.2720	0.097*	
C15	0.8049 (2)	0.8418 (2)	0.2685 (2)	0.0587 (6)	
H15	0.8931	0.8022	0.2727	0.070*	
C16	0.58220 (13)	0.58587 (12)	0.10469 (13)	0.0292 (3)	
H16A	0.5727	0.5253	0.1792	0.035*	
H16B	0.5688	0.5514	0.0504	0.035*	
C17	0.46644 (13)	0.68702 (12)	0.12265 (12)	0.0269 (2)	
C18	0.47327 (14)	0.79830 (13)	0.04363 (13)	0.0312 (3)	
H18	0.5530	0.8133	−0.0208	0.037*	
C19	0.36222 (16)	0.88845 (13)	0.05909 (14)	0.0347 (3)	
H19	0.3682	0.9628	0.0047	0.042*	
C20	0.24365 (15)	0.86818 (14)	0.15431 (15)	0.0350 (3)	
C21	0.23710 (14)	0.75648 (14)	0.23783 (13)	0.0326 (3)	
C22	0.34699 (14)	0.66710 (13)	0.22105 (12)	0.0296 (3)	
H22	0.3417	0.5928	0.2756	0.036*	
C23	0.1240 (3)	1.0559 (2)	0.0848 (3)	0.0857 (10)	
H23A	0.1932	1.1019	0.0787	0.129*	
H23B	0.0323	1.1002	0.1029	0.129*	
H23C	0.1440	1.0385	0.0115	0.129*	
C24	0.1089 (2)	0.6366 (2)	0.42176 (19)	0.0577 (5)	
H24A	0.1140	0.5729	0.3903	0.087*	
H24B	0.0213	0.6415	0.4826	0.087*	
H24C	0.1855	0.6224	0.4539	0.087*	
C25	0.79102 (15)	0.63214 (13)	−0.15877 (13)	0.0311 (3)	
H25	0.8700	0.6102	−0.2158	0.037*	
C26	0.67095 (15)	0.65761 (15)	−0.18607 (13)	0.0342 (3)	
H26	0.5864	0.6751	−0.1316	0.041*	
C27	0.67332 (16)	0.65776 (16)	−0.30294 (13)	0.0358 (3)	
C28	0.5395 (2)	0.6890 (3)	−0.43115 (17)	0.0634 (6)	
H28A	0.5753	0.6117	−0.4448	0.076*	
H28B	0.5976	0.7465	−0.4927	0.076*	
C29	0.3931 (3)	0.7211 (3)	−0.4337 (2)	0.0673 (7)	
H29A	0.3356	0.6655	−0.3710	0.101*	
H29B	0.3881	0.7196	−0.5077	0.101*	
H29C	0.3599	0.7993	−0.4242	0.101*	
N1	0.80387 (12)	0.62707 (11)	0.13020 (10)	0.0292 (2)	
O1	0.61261 (13)	0.58330 (11)	0.31986 (10)	0.0420 (3)	
O2	0.85541 (15)	0.58180 (14)	0.31850 (11)	0.0509 (3)	
O3	0.12766 (14)	0.94873 (12)	0.17533 (14)	0.0550 (4)	
O4	0.11775 (12)	0.74569 (12)	0.33019 (12)	0.0468 (3)	
O7	0.77576 (14)	0.63380 (17)	−0.37800 (13)	0.0608 (4)	
O8	0.54527 (12)	0.68657 (14)	−0.31723 (10)	0.0482 (3)	
S1	0.74189 (4)	0.63276 (4)	0.26998 (3)	0.03379 (10)	
C30	1.0269 (8)	−0.0254 (6)	0.4558 (6)	0.113 (3)	0.50
H30A	1.0428	−0.0660	0.3979	0.136*	0.50
Cl1	0.9317 (6)	0.1115 (4)	0.4302 (5)	0.199 (2)	0.50
Cl2	0.9683 (8)	−0.1068 (4)	0.5950 (4)	0.223 (3)	0.50
Cl3	1.1858 (5)	0.0222 (6)	0.4566 (6)	0.241 (2)	0.50

### Atomic displacement parameters (Å^2^)

**Table:**

	*U*^11^	*U*^22^	*U*^33^	*U*^12^	*U*^13^	*U*^23^
C2	0.0239 (5)	0.0266 (6)	0.0284 (6)	−0.0029 (4)	−0.0068 (4)	−0.0083 (5)
C3	0.0237 (5)	0.0287 (6)	0.0294 (6)	−0.0024 (4)	−0.0067 (4)	−0.0093 (5)
C4	0.0233 (5)	0.0246 (6)	0.0297 (6)	−0.0015 (4)	−0.0063 (4)	−0.0084 (5)
C5	0.0256 (6)	0.0342 (7)	0.0311 (6)	−0.0035 (5)	−0.0032 (5)	−0.0102 (5)
C6	0.0237 (6)	0.0394 (8)	0.0433 (8)	−0.0042 (5)	−0.0032 (5)	−0.0149 (6)
C7	0.0250 (6)	0.0452 (8)	0.0483 (9)	−0.0049 (5)	−0.0097 (6)	−0.0199 (7)
C8	0.0286 (6)	0.0450 (8)	0.0383 (8)	−0.0033 (5)	−0.0102 (5)	−0.0186 (6)
C9	0.0242 (5)	0.0283 (6)	0.0311 (6)	−0.0017 (4)	−0.0072 (4)	−0.0102 (5)
C10	0.0395 (7)	0.0459 (8)	0.0285 (7)	−0.0127 (6)	−0.0010 (5)	−0.0162 (6)
C11	0.0526 (9)	0.0454 (9)	0.0356 (8)	−0.0058 (7)	−0.0139 (7)	−0.0158 (7)
C12	0.0707 (13)	0.0472 (11)	0.0538 (12)	−0.0002 (9)	−0.0168 (10)	−0.0197 (9)
C13	0.0876 (18)	0.0528 (13)	0.0797 (18)	−0.0212 (12)	0.0009 (14)	−0.0335 (12)
C14	0.0689 (15)	0.0828 (18)	0.102 (2)	−0.0386 (14)	0.0080 (14)	−0.0570 (17)
C15	0.0406 (9)	0.0773 (15)	0.0694 (14)	−0.0203 (9)	0.0008 (8)	−0.0441 (12)
C16	0.0246 (5)	0.0294 (6)	0.0327 (6)	−0.0057 (4)	−0.0051 (5)	−0.0095 (5)
C17	0.0223 (5)	0.0303 (6)	0.0278 (6)	−0.0055 (4)	−0.0055 (4)	−0.0081 (5)
C18	0.0268 (6)	0.0339 (7)	0.0293 (6)	−0.0069 (5)	−0.0041 (5)	−0.0066 (5)
C19	0.0325 (6)	0.0293 (7)	0.0364 (7)	−0.0056 (5)	−0.0072 (5)	−0.0038 (5)
C20	0.0287 (6)	0.0310 (7)	0.0409 (8)	−0.0015 (5)	−0.0059 (5)	−0.0104 (6)
C21	0.0250 (6)	0.0362 (7)	0.0332 (7)	−0.0061 (5)	−0.0021 (5)	−0.0104 (6)
C22	0.0260 (5)	0.0300 (6)	0.0296 (6)	−0.0064 (4)	−0.0052 (5)	−0.0055 (5)
C23	0.0520 (12)	0.0391 (11)	0.113 (2)	0.0090 (9)	0.0024 (13)	0.0079 (12)
C24	0.0444 (9)	0.0549 (11)	0.0452 (10)	−0.0098 (8)	0.0117 (8)	−0.0017 (8)
C25	0.0298 (6)	0.0364 (7)	0.0278 (6)	−0.0047 (5)	−0.0068 (5)	−0.0110 (5)
C26	0.0302 (6)	0.0446 (8)	0.0295 (6)	−0.0017 (5)	−0.0078 (5)	−0.0156 (6)
C27	0.0322 (6)	0.0477 (8)	0.0293 (7)	−0.0073 (6)	−0.0066 (5)	−0.0141 (6)
C28	0.0493 (10)	0.116 (2)	0.0294 (8)	−0.0157 (11)	−0.0110 (7)	−0.0228 (10)
C29	0.0630 (13)	0.103 (2)	0.0438 (11)	−0.0165 (13)	−0.0262 (10)	−0.0151 (12)
N1	0.0253 (5)	0.0349 (6)	0.0271 (5)	−0.0041 (4)	−0.0054 (4)	−0.0106 (4)
O1	0.0429 (6)	0.0477 (7)	0.0297 (5)	−0.0165 (5)	−0.0017 (4)	−0.0060 (5)
O2	0.0485 (7)	0.0687 (9)	0.0352 (6)	−0.0030 (6)	−0.0203 (5)	−0.0084 (6)
O3	0.0369 (6)	0.0367 (6)	0.0681 (9)	0.0056 (5)	0.0009 (6)	−0.0092 (6)
O4	0.0319 (5)	0.0444 (7)	0.0440 (7)	−0.0035 (5)	0.0078 (5)	−0.0077 (5)
O7	0.0395 (7)	0.1053 (13)	0.0413 (7)	−0.0004 (7)	−0.0046 (5)	−0.0383 (8)
O8	0.0346 (6)	0.0831 (10)	0.0283 (5)	−0.0080 (6)	−0.0089 (4)	−0.0174 (6)
S1	0.03402 (18)	0.0409 (2)	0.02481 (17)	−0.00679 (14)	−0.00670 (12)	−0.00787 (14)
C30	0.145 (6)	0.099 (5)	0.098 (5)	0.038 (4)	−0.053 (4)	−0.047 (4)
Cl1	0.273 (5)	0.147 (3)	0.235 (5)	0.091 (3)	−0.201 (5)	−0.071 (3)
Cl2	0.327 (6)	0.129 (3)	0.147 (3)	−0.070 (4)	0.001 (4)	−0.003 (2)
Cl3	0.162 (3)	0.292 (6)	0.246 (5)	0.010 (4)	−0.066 (3)	−0.065 (4)

### Geometric parameters (Å, °)

**Table:**

C2—C3	1.3669 (19)	C23—O3	1.416 (3)
C2—N1	1.4146 (17)	C23—H23A	0.9600
C2—C16	1.4948 (17)	C23—H23B	0.9600
C3—C4	1.4424 (17)	C23—H23C	0.9600
C3—C25	1.4503 (19)	C24—O4	1.430 (2)
C4—C5	1.3932 (18)	C24—H24A	0.9600
C4—C9	1.3956 (19)	C24—H24B	0.9600
C5—C6	1.385 (2)	C24—H24C	0.9600
C5—H5	0.9300	C25—C26	1.332 (2)
C6—C7	1.387 (2)	C25—H25	0.9300
C6—H6	0.9300	C26—C27	1.467 (2)
C7—C8	1.381 (2)	C26—H26	0.9300
C7—H7	0.9300	C27—O7	1.1969 (19)
C8—C9	1.3931 (19)	C27—O8	1.3355 (19)
C8—H8	0.9300	C28—O8	1.451 (2)
C9—N1	1.4148 (17)	C28—C29	1.467 (3)
C10—C11	1.384 (3)	C28—H28A	0.9700
C10—C15	1.385 (2)	C28—H28B	0.9700
C10—S1	1.7581 (18)	C29—H29A	0.9600
C11—C12	1.381 (3)	C29—H29B	0.9600
C11—H11	0.9300	C29—H29C	0.9600
C12—C13	1.377 (4)	N1—S1	1.6749 (12)
C12—H12	0.9300	O1—S1	1.4211 (12)
C13—C14	1.366 (4)	O2—S1	1.4225 (13)
C13—H13	0.9300	C30—C30^i^	1.341 (14)
C14—C15	1.387 (4)	C30—Cl2^i^	1.512 (8)
C14—H14	0.9300	C30—Cl1^i^	1.601 (8)
C15—H15	0.9300	C30—Cl2	1.673 (7)
C16—C17	1.5121 (19)	C30—Cl1	1.708 (6)
C16—H16A	0.9700	C30—Cl3	1.842 (8)
C16—H16B	0.9700	C30—Cl3^i^	2.065 (10)
C17—C18	1.380 (2)	C30—H30A	0.9600
C17—C22	1.4028 (18)	Cl1—Cl2^i^	0.952 (7)
C18—C19	1.393 (2)	Cl1—C30^i^	1.601 (8)
C18—H18	0.9300	Cl1—Cl3^i^	2.054 (8)
C19—C20	1.377 (2)	Cl2—Cl1^i^	0.952 (7)
C19—H19	0.9300	Cl2—C30^i^	1.512 (8)
C20—O3	1.3613 (19)	Cl2—Cl3^i^	1.871 (8)
C20—C21	1.406 (2)	Cl3—Cl2^i^	1.871 (8)
C21—O4	1.3577 (17)	Cl3—Cl1^i^	2.054 (8)
C21—C22	1.382 (2)	Cl3—C30^i^	2.065 (10)
C22—H22	0.9300		
			
C3—C2—N1	108.14 (11)	C26—C25—H25	115.1
C3—C2—C16	127.64 (12)	C3—C25—H25	115.1
N1—C2—C16	123.93 (12)	C25—C26—C27	119.37 (13)
C2—C3—C4	108.30 (12)	C25—C26—H26	120.3
C2—C3—C25	129.64 (12)	C27—C26—H26	120.3
C4—C3—C25	122.05 (12)	O7—C27—O8	122.46 (15)
C5—C4—C9	119.96 (12)	O7—C27—C26	125.42 (15)
C5—C4—C3	132.26 (13)	O8—C27—C26	112.11 (13)
C9—C4—C3	107.78 (11)	O8—C28—C29	109.08 (17)
C6—C5—C4	118.49 (14)	O8—C28—H28A	109.9
C6—C5—H5	120.8	C29—C28—H28A	109.9
C4—C5—H5	120.8	O8—C28—H28B	109.9
C5—C6—C7	120.58 (13)	C29—C28—H28B	109.9
C5—C6—H6	119.7	H28A—C28—H28B	108.3
C7—C6—H6	119.7	C28—C29—H29A	109.5
C8—C7—C6	122.15 (14)	C28—C29—H29B	109.5
C8—C7—H7	118.9	H29A—C29—H29B	109.5
C6—C7—H7	118.9	C28—C29—H29C	109.5
C7—C8—C9	116.95 (14)	H29A—C29—H29C	109.5
C7—C8—H8	121.5	H29B—C29—H29C	109.5
C9—C8—H8	121.5	C2—N1—C9	108.64 (11)
C8—C9—C4	121.85 (13)	C2—N1—S1	128.29 (9)
C8—C9—N1	131.06 (13)	C9—N1—S1	122.44 (10)
C4—C9—N1	107.10 (11)	C20—O3—C23	116.43 (16)
C11—C10—C15	121.11 (18)	C21—O4—C24	117.78 (14)
C11—C10—S1	118.80 (13)	C27—O8—C28	115.21 (14)
C15—C10—S1	120.02 (16)	O1—S1—O2	120.63 (8)
C12—C11—C10	119.32 (19)	O1—S1—N1	106.73 (7)
C12—C11—H11	120.3	O2—S1—N1	105.65 (7)
C10—C11—H11	120.3	O1—S1—C10	108.29 (8)
C13—C12—C11	120.1 (2)	O2—S1—C10	108.90 (9)
C13—C12—H12	120.0	N1—S1—C10	105.65 (7)
C11—C12—H12	120.0	C30^i^—C30—Cl2^i^	71.5 (6)
C14—C13—C12	120.2 (2)	C30^i^—C30—Cl1^i^	70.3 (6)
C14—C13—H13	119.9	Cl2^i^—C30—Cl1^i^	128.4 (6)
C12—C13—H13	119.9	C30^i^—C30—Cl2	59.0 (5)
C13—C14—C15	121.2 (2)	Cl2^i^—C30—Cl2	130.5 (5)
C13—C14—H14	119.4	Cl1^i^—C30—Cl2	33.7 (3)
C15—C14—H14	119.4	C30^i^—C30—Cl1	62.0 (5)
C10—C15—C14	118.2 (2)	Cl2^i^—C30—Cl1	33.7 (3)
C10—C15—H15	120.9	Cl1^i^—C30—Cl1	132.3 (4)
C14—C15—H15	120.9	Cl2—C30—Cl1	112.0 (4)
C2—C16—C17	115.26 (11)	C30^i^—C30—Cl3	79.2 (7)
C2—C16—H16A	108.5	Cl2^i^—C30—Cl3	67.0 (4)
C17—C16—H16A	108.5	Cl1^i^—C30—Cl3	72.9 (4)
C2—C16—H16B	108.5	Cl2—C30—Cl3	101.7 (5)
C17—C16—H16B	108.5	Cl1—C30—Cl3	97.4 (4)
H16A—C16—H16B	107.5	C30^i^—C30—Cl3^i^	61.2 (6)
C18—C17—C22	118.73 (13)	Cl2^i^—C30—Cl3^i^	98.2 (4)
C18—C17—C16	121.92 (12)	Cl1^i^—C30—Cl3^i^	92.5 (4)
C22—C17—C16	119.34 (12)	Cl2—C30—Cl3^i^	59.0 (4)
C17—C18—C19	120.84 (13)	Cl1—C30—Cl3^i^	65.2 (3)
C17—C18—H18	119.6	Cl3—C30—Cl3^i^	140.4 (4)
C19—C18—H18	119.6	C30^i^—C30—H30A	166.2
C20—C19—C18	120.44 (14)	Cl2^i^—C30—H30A	113.6
C20—C19—H19	119.8	Cl1^i^—C30—H30A	111.7
C18—C19—H19	119.8	Cl2—C30—H30A	114.6
O3—C20—C19	125.06 (15)	Cl1—C30—H30A	114.5
O3—C20—C21	115.52 (13)	Cl3—C30—H30A	114.6
C19—C20—C21	119.42 (13)	Cl3^i^—C30—H30A	105.0
O4—C21—C22	125.01 (14)	Cl2^i^—Cl1—C30^i^	77.3 (4)
O4—C21—C20	115.26 (13)	Cl2^i^—Cl1—C30	61.8 (5)
C22—C21—C20	119.73 (13)	C30^i^—Cl1—C30	47.7 (4)
C21—C22—C17	120.79 (13)	Cl2^i^—Cl1—Cl3^i^	126.3 (5)
C21—C22—H22	119.6	C30^i^—Cl1—Cl3^i^	59.0 (4)
C17—C22—H22	119.6	C30—Cl1—Cl3^i^	65.8 (3)
O3—C23—H23A	109.5	Cl1^i^—Cl2—C30^i^	84.5 (6)
O3—C23—H23B	109.5	Cl1^i^—Cl2—C30	69.0 (5)
H23A—C23—H23B	109.5	C30^i^—Cl2—C30	49.5 (5)
O3—C23—H23C	109.5	Cl1^i^—Cl2—Cl3^i^	139.6 (5)
H23A—C23—H23C	109.5	C30^i^—Cl2—Cl3^i^	65.0 (4)
H23B—C23—H23C	109.5	C30—Cl2—Cl3^i^	71.0 (4)
O4—C24—H24A	109.5	C30—Cl3—Cl2^i^	48.0 (3)
O4—C24—H24B	109.5	C30—Cl3—Cl1^i^	48.2 (3)
H24A—C24—H24B	109.5	Cl2^i^—Cl3—Cl1^i^	91.0 (3)
O4—C24—H24C	109.5	C30—Cl3—C30^i^	39.6 (4)
H24A—C24—H24C	109.5	Cl2^i^—Cl3—C30^i^	50.0 (2)
H24B—C24—H24C	109.5	Cl1^i^—Cl3—C30^i^	49.0 (2)
C26—C25—C3	129.71 (14)		
			
N1—C2—C3—C4	0.77 (15)	O7—C27—O8—C28	−1.2 (3)
C16—C2—C3—C4	−173.17 (13)	C26—C27—O8—C28	179.76 (18)
N1—C2—C3—C25	179.76 (14)	C29—C28—O8—C27	179.7 (2)
C16—C2—C3—C25	5.8 (2)	C2—N1—S1—O1	21.70 (15)
C2—C3—C4—C5	178.86 (15)	C9—N1—S1—O1	−168.44 (11)
C25—C3—C4—C5	−0.2 (2)	C2—N1—S1—O2	151.24 (13)
C2—C3—C4—C9	−1.78 (15)	C9—N1—S1—O2	−38.90 (14)
C25—C3—C4—C9	179.13 (13)	C2—N1—S1—C10	−93.43 (13)
C9—C4—C5—C6	−1.5 (2)	C9—N1—S1—C10	76.43 (13)
C3—C4—C5—C6	177.80 (15)	C11—C10—S1—O1	−25.83 (15)
C4—C5—C6—C7	0.1 (2)	C15—C10—S1—O1	151.37 (15)
C5—C6—C7—C8	1.2 (3)	C11—C10—S1—O2	−158.70 (13)
C6—C7—C8—C9	−1.0 (2)	C15—C10—S1—O2	18.50 (18)
C7—C8—C9—C4	−0.4 (2)	C11—C10—S1—N1	88.22 (14)
C7—C8—C9—N1	179.80 (15)	C15—C10—S1—N1	−94.58 (16)
C5—C4—C9—C8	1.7 (2)	C30^i^—C30—Cl1—Cl2^i^	98.6 (8)
C3—C4—C9—C8	−177.79 (13)	Cl1^i^—C30—Cl1—Cl2^i^	98.6 (8)
C5—C4—C9—N1	−178.49 (12)	Cl2—C30—Cl1—Cl2^i^	131.0 (6)
C3—C4—C9—N1	2.06 (15)	Cl3—C30—Cl1—Cl2^i^	25.1 (6)
C15—C10—C11—C12	−0.1 (3)	Cl3^i^—C30—Cl1—Cl2^i^	167.8 (7)
S1—C10—C11—C12	177.03 (15)	Cl2^i^—C30—Cl1—C30^i^	−98.6 (8)
C10—C11—C12—C13	0.3 (3)	Cl1^i^—C30—Cl1—C30^i^	0.0
C11—C12—C13—C14	−0.4 (4)	Cl2—C30—Cl1—C30^i^	32.3 (5)
C12—C13—C14—C15	0.3 (5)	Cl3—C30—Cl1—C30^i^	−73.5 (6)
C11—C10—C15—C14	0.1 (3)	Cl3^i^—C30—Cl1—C30^i^	69.2 (6)
S1—C10—C15—C14	−177.1 (2)	C30^i^—C30—Cl1—Cl3^i^	−69.2 (6)
C13—C14—C15—C10	−0.2 (4)	Cl2^i^—C30—Cl1—Cl3^i^	−167.8 (7)
C3—C2—C16—C17	−103.22 (16)	Cl1^i^—C30—Cl1—Cl3^i^	−69.2 (6)
N1—C2—C16—C17	83.73 (16)	Cl2—C30—Cl1—Cl3^i^	−36.8 (5)
C2—C16—C17—C18	40.18 (19)	Cl3—C30—Cl1—Cl3^i^	−142.7 (4)
C2—C16—C17—C22	−141.19 (13)	C30^i^—C30—Cl2—Cl1^i^	−101.1 (8)
C22—C17—C18—C19	−1.8 (2)	Cl2^i^—C30—Cl2—Cl1^i^	−101.1 (8)
C16—C17—C18—C19	176.82 (13)	Cl1—C30—Cl2—Cl1^i^	−134.6 (6)
C17—C18—C19—C20	0.4 (2)	Cl3—C30—Cl2—Cl1^i^	−31.5 (6)
C18—C19—C20—O3	−177.63 (16)	Cl3^i^—C30—Cl2—Cl1^i^	−174.0 (7)
C18—C19—C20—C21	1.8 (2)	Cl2^i^—C30—Cl2—C30^i^	0.0
O3—C20—C21—O4	−2.2 (2)	Cl1^i^—C30—Cl2—C30^i^	101.1 (8)
C19—C20—C21—O4	178.36 (15)	Cl1—C30—Cl2—C30^i^	−33.4 (4)
O3—C20—C21—C22	176.88 (15)	Cl3—C30—Cl2—C30^i^	69.6 (6)
C19—C20—C21—C22	−2.6 (2)	Cl3^i^—C30—Cl2—C30^i^	−72.9 (6)
O4—C21—C22—C17	−179.85 (14)	C30^i^—C30—Cl2—Cl3^i^	72.9 (6)
C20—C21—C22—C17	1.2 (2)	Cl2^i^—C30—Cl2—Cl3^i^	72.9 (6)
C18—C17—C22—C21	1.0 (2)	Cl1^i^—C30—Cl2—Cl3^i^	174.0 (7)
C16—C17—C22—C21	−177.68 (13)	Cl1—C30—Cl2—Cl3^i^	39.4 (5)
C2—C3—C25—C26	26.6 (3)	Cl3—C30—Cl2—Cl3^i^	142.5 (4)
C4—C3—C25—C26	−154.55 (16)	C30^i^—C30—Cl3—Cl2^i^	−74.4 (5)
C3—C25—C26—C27	175.66 (15)	Cl1^i^—C30—Cl3—Cl2^i^	−146.9 (5)
C25—C26—C27—O7	1.8 (3)	Cl2—C30—Cl3—Cl2^i^	−129.3 (5)
C25—C26—C27—O8	−179.10 (16)	Cl1—C30—Cl3—Cl2^i^	−14.8 (3)
C3—C2—N1—C9	0.52 (15)	Cl3^i^—C30—Cl3—Cl2^i^	−74.4 (5)
C16—C2—N1—C9	174.73 (12)	C30^i^—C30—Cl3—Cl1^i^	72.6 (5)
C3—C2—N1—S1	171.49 (10)	Cl2^i^—C30—Cl3—Cl1^i^	146.9 (5)
C16—C2—N1—S1	−14.3 (2)	Cl2—C30—Cl3—Cl1^i^	17.7 (3)
C8—C9—N1—C2	178.21 (15)	Cl1—C30—Cl3—Cl1^i^	132.1 (4)
C4—C9—N1—C2	−1.62 (15)	Cl3^i^—C30—Cl3—Cl1^i^	72.6 (5)
C8—C9—N1—S1	6.6 (2)	Cl2^i^—C30—Cl3—C30^i^	74.4 (5)
C4—C9—N1—S1	−173.23 (10)	Cl1^i^—C30—Cl3—C30^i^	−72.6 (5)
C19—C20—O3—C23	7.7 (3)	Cl2—C30—Cl3—C30^i^	−54.9 (5)
C21—C20—O3—C23	−171.8 (2)	Cl1—C30—Cl3—C30^i^	59.5 (5)
C22—C21—O4—C24	4.9 (3)	Cl3^i^—C30—Cl3—C30^i^	0.000 (1)
C20—C21—O4—C24	−176.06 (18)		

Symmetry codes: (i) −*x*+2, −*y*, −*z*+1.

### Hydrogen-bond geometry (Å, °)

**Table:**

*D*—H···*A*	*D*—H	H···*A*	*D*···*A*	*D*—H···*A*
C8—H8···O2	0.93	2.38	2.928 (2)	118
C15—H15···O4^ii^	0.93	2.53	3.411 (3)	159
C16—H16A···O1	0.97	2.30	2.831 (2)	114
C24—H24B···O7^iii^	0.96	2.51	3.461 (2)	169
C24—H24A···O7^iv^	0.96	2.52	3.367 (3)	147
C25—H25···O7	0.93	2.46	2.821 (2)	103
C30—H30A···O3^v^	0.96	2.59	3.429 (7)	146
C30—H30A···O4^v^	0.96	2.55	3.424 (7)	151

Symmetry codes: (ii) *x*+1, *y*, *z*; (iii) *x*−1, *y*, *z*+1; (iv) −*x*+1, −*y*+1, −*z*; (v) *x*+1, *y*−1, *z*.
